# Catastrophic Health Expenditures and Their Determinants Among Stroke Patients and Their Households: A Study From Semnan, Iran

**DOI:** 10.1002/hsr2.71481

**Published:** 2025-11-10

**Authors:** Sayed Amir Mortazavi, Sayed Saeed Kassaeian, Navid Danaei, Farid Gharibi

**Affiliations:** ^1^ School of Medicine Semnan University of Medical Sciences Semnan Iran; ^2^ Department of Community Medicine School of Medicine Semnan University of Medical Sciences Semnan Iran; ^3^ Department of Pediatrics School of Medicine Semnan University of Medical Sciences Semnan Iran; ^4^ Social Determinants of Health Research Center Semnan University of Medical Sciences Semnan Iran; ^5^ Department of Health Services Management School of Nursing and Midwifery Semnan University of Medical Sciences Semnan Iran

**Keywords:** catastrophic health expenditures, equity, financial protection, stroke

## Abstract

**Background:**

Stroke sufferers may be exposed to unmanageable medical costs because of the severe complications that can arise from the condition and the impairment of their economic capacity.

**Objective:**

This study investigates the prevalence and underlying factors of catastrophic health expenditure (CHE) among stroke patients in Semnan, Iran.

**Methods:**

The present cross‐sectional study was conducted in 2024 with the participation of 270 patients. The study tool was a researcher‐designed questionnaire based on patients' clinical records. The content validity of the questionnaire was subsequently confirmed by experts, obtaining scores of 0.96 and 0.94 for the CVR and CVI indices, respectively. The incidence of CHE was calculated using the model of “allocating at least 40% of household non‐food expenses for health care.” The statistical relationship between demographic and background variables and the occurrence of CHE was assessed using logistic regression.

**Results:**

Stroke patients annually pay an average of 339,580,000 IRR (915.86 USD) for necessary care, with 5% allocated to diagnostic services, 12% to physician visits, and 83% to treatment care. The ratio of direct medical costs to non‐food expenses was estimated at 1.30 ( ± 2.30), and 67% of patients face CHE. In the regression analyses, variables such as age (OR: 1.572), supplemental insurance coverage (OR: 2.501), and the occurrence of disease complications such as facial paralysis (OR: 0.253), limb paralysis (OR: 0.122), and impaired consciousness (OR: 0.228) were identified as influencing factors for CHE (*p* < 0.05).

**Conclusions:**

The high costs imposed on patients with strokes, especially in terms of medications and hospitalization, have imposed unusual economic pressure on patients and the occurrence of an unusual and unacceptable level of CHE. The study also identified some demographic and background determinants of the occurrence of CHE in patients with stroke, which should be noted in conducting interventions by policymakers.

## 1 Introduction

Stroke, caused by the lack of blood supply to a part of the brain due to blockage or rupture of cerebral vessels, is the second leading cause of death and one of the primary causes of long‐term disability worldwide [1]. Annually, 15 million stroke cases occur globally, with one‐third resulting in death and another one‐third leading to permanent disabilities [2]. In 2017 alone, approximately 1.5 million people in European countries experienced a stroke, 9 million people faced its devastating effects, and 438,000 patients died [3]. In 2013, stroke was the leading cause of death in most parts of China, which has the highest stroke prevalence in the world at 7.2% [4].

The annual incidence of stroke in Iran varies between 23 and 103 cases per 100,000 people [5]. This rate is similar to that of Arab countries and most sub‐Saharan African countries, but lower than that of developing countries, India, Latin America, and China [5]. A concerning trend is that the prevalence of stroke in low‐ and middle‐income countries has increased by more than 20% over the past decade [6]. In Iran, the prevalence has risen from 84.16 to 103.23 cases per 100,000 people over 4 years [5]. Additionally, the age of stroke onset in Iran is about 10 years younger than in many developing countries, and the mortality rate is higher [7]. Overall, stroke accounts for approximately 11% of all deaths in Iran, with an annual increase of 1% in this statistic. Moreover, the disease contributes to 1.56% of years lived with disability (YLDs) and 4.48% of the disability‐adjusted life years (DALYs) in Iran [8].

The global prevalence of stroke is projected to continue rising due to the ongoing phenomenon of population aging and the increasing prevalence of hypertension and obesity [9]. Additionally, the reduction in stroke‐related mortality, coupled with the growing proportion of elderly populations in various societies, implies an upward trend in long‐term disabilities [2]. Therefore, the high burden of this disease, due to its prevalence, hospitalization rate, mortality, and severe complications, is profoundly significant [10]. Stroke patients utilize healthcare services more than others, incur higher care costs, and have lower employment rates [11]. Moreover, stroke survivors often require long‐term medical and personal care due to the complications and disabilities resulting from the stroke [12]. Thus, stroke not only poses a significant public health issue but also has extensive economic and social impacts on households, the healthcare system, and societies [13].

Stroke alone accounts for 2%–4% of all healthcare expenditures worldwide [1]. In 2017, it imposed a cost of 60 billion euros on European countries, with 27 billion euros attributed to healthcare expenses. The four most populous European countries—Germany, France, Italy, and the United Kingdom—bore 63% of these costs [3]. In 2017, approximately 43 million days of nursing care and long‐term care center services were provided due to stroke, resulting in an additional 4.7 billion euros in costs to the European social care system [3]. In the United States, direct medical costs of stroke amounted to 22.8 billion dollars in 2009, while in Iceland, Norway, and Switzerland, these costs reached 26.6 billion dollars in 2010 [2]. Another concerning trend is the increasing costs associated with stroke due to the growing elderly population and the escalating complexity and expense of care [3]. In the United States, direct medical costs for stroke treatment rose from 38 billion dollars in 2015 to 51.3 billion dollars in 2020 [14], and it is projected that annual stroke costs in the United States will increase by 129% to 240.67 billion dollars by 2030 [15].

The point worth contemplating is the necessity to investigate the costs imposed on patients and their families and assess the appropriateness of these costs relative to their ability to pay. Financial protection of citizens against excessive healthcare costs is a societal responsibility. The inability of patients and their families to afford healthcare expenses can lead them to forgo care or delay it. Continuation of these conditions can result in deteriorating health outcomes, reduced satisfaction, and a noticeable decline in the living standards of patients and their families [16]. Given that these conditions often prevail in chronic diseases, which not only incur high care costs but also have disabling implications that diminish patients' economic capacity [17], this study was conducted to examine the occurrence and influencing factors of CHE in stroke patients in Semnan City.

## 2 Methods

### 2.1 Study Design and Participants

The cross‐sectional study was conducted in 2024 with the participation of stroke patients. Given the need to examine costs over 1 year, the inclusion criteria for patients in the study included at least 1 year after the stroke and receiving care during this period. The exclusion criteria for patients from the study also included the absence of other serious and related diseases, such as Ischemic Heart Disease (IHD) and Chronic Kidney Disease. Patients exited the survey if they were unable to respond due to health conditions, if it was not possible to obtain information from primary caregivers, or in the event of their death before the study could be completed.

### 2.2 Sample Size

The sample size for the study was determined based on the following formula:

$$n={\left(\frac{\mathrm{zs}}{d}\right)}^{2}={\left(\frac{1.96*0.5}{0.05}\right)}^{2}=\left(\frac{0.9604}{0.0025}\right)=384.16$$

This formula specifies the value of the Z‐score at a confidence level of z, the expected changes in responses with s, and the margin of error with d. Therefore, the sample size for this study was determined to be 384 cases at a 95% confidence level, with an expected change of 0.05 and a margin of error of 0.05. However, due to the limited number of cases in Semnan, 270 patients were examined.

### 2.3 Data Collection Tools

To develop and standardize the questionnaire, the cost components related to stroke were initially identified through interviews with health economics and management specialists, neurologists, patient interviews, a review of their medical records, and a literature review of similar studies. Next, demographic and background variables potentially influencing costs and their catastrophic nature were determined based on experts' perspectives, and similar studies were assessed and formed the initial questionnaire. Subsequently, the content and face validity of the questionnaire were evaluated based on the opinions of 10 experts. The questions in the initial questionnaire were evaluated across four criteria: necessity, relevance, clarity, and simplicity. Content Validity Ratio (CVR) and Content Validity Index (CVI) were calculated [16]. The face validity was also evaluated by obtaining expert feedback on how the questionnaire content was drafted. The results for each item in the necessity criterion were used to determine the CVR index. For calculating the CVI index, the results obtained from three other criteria were employed according to the following formula [16]:

$$\text{CVR}=\frac{\text{nE}-\frac{N}{2}}{\frac{N}{2}}$$

nE represents the number of experts selecting items with positive ratings, and N is the total number of experts. The questionnaire underwent psychometric validation with the participation of 10 experts. Due to their involvement, a criterion of 0.62 acceptance score was set [16]. The final questionnaire comprised 62 questions, with 21 questions relating to demographic/background variables, 37 questions addressing cost dimensions, and four questions concerning household income and expenditure methods. This study examined the patients' exposure to stroke complications using five questions in the background variables section. These complications included facial paralysis, limb paralysis, speech disorders, impaired consciousness, and other complications (numbness of hands and feet, bleeding eyes, etc.). The validity of the final tool was confirmed with acceptance scores of 0.96 for CVR and 0.94 for CVI, based on expert evaluations.

### 2.4 The Location and Method of Data Collection

The study was conducted at healthcare facilities in Semnan city, including Kowsar Hospital and the Rehabilitation Clinic affiliated with the Rehabilitation School. The required data for the study were collected through in‐person interviews conducted at these healthcare facilities and telephone calls with patients. Additionally, the clinical documents of the patients were reviewed to verify their statements as much as possible, ensuring the accuracy of the data.

### 2.5 Data Analysis

Studies indicate that CHE refers to healthcare costs exceeding a patient's ability to pay [18]. When healthcare expenditures by patients and their households surpass 40% of their non‐food household expenses, it signifies the occurrence of CHE [18]. To examine healthcare costs and their catastrophic nature, data on expenses and incomes of patients from the past year were collected and analyzed. Data collection involved both face‐to‐face and telephone interviews with patients. Each patient was asked questions, and the interviewer recorded their responses in the relevant questionnaire. The obtained healthcare costs are based on the exchange rates of the Central Bank of Iran, expressed in US dollars (a USD equivalent to 370,777 IRR at 2024 June 4). Subsequently, the proportion of direct medical expenses relative to non‐food household expenses was calculated, reaching a minimum threshold of 40% to define CHE. Univariate (non‐adjusted) and multivariate (adjusted) logistic regression analyses were employed to investigate the statistical relationship between demographic and background variables and the occurrence of CHE. The pairwise statistical relationships between demographic and background variables with CHE were initially examined. The multivariable analyses and modeling included variables showing a significance level of less than 0.20. Further refinement was conducted using backward LR to identify the final set of variables significantly associated with CHE.

### 2.6 Ethical Considerations

Researchers adhered to ethical principles such as ensuring patient freedom to participate in the study, obtaining informed consent, ensuring patient anonymity, and using acquired information exclusively for the study's objectives. They took precautions to avoid disrupting patient treatment processes during data collection and obtained ethical approval (IR.SEMUMS.REC.1400.210) to conduct the research study.

## 3 Results

The average age of the patients under study was 65.05 years ( ± 13.04 years). Approximately 13% were under 50, 17% were aged between 50 and 59, and more than 70% were elderly. The distribution of disease prevalence between the two genders was nearly equal, with two‐thirds of the patients being married. Less than a quarter of the patients (23%) had a university education, and one‐third were illiterate. Retired individuals (41%) and homemakers (32%) comprised the largest occupational groups. More than three‐quarters (78%) of the patients lived in urban areas, with over half (56%) residing in the provincial center. Most (98%) of patients had basic health insurance, with three‐quarters (75%) covered by social insurance. Additionally, over two‐thirds (71%) had supplemental health insurance (Table 1).

**TABLE 1:** Demographic and background characteristics of patients.

Demographic characteristics	Grouping	Frequency	Percentage
Age	Less than 40 years	13	4.8
	40–49 years	21	7.8
	50–59 years	46	17
	60–69 years	79	29.3
	70–79 years	81	30
	80 years and older	30	11.1
Gender	Female	124	45.9
	Man	146	54.1
Marital status	Single	10	3.7
	Married	179	66.3
	Isolated	5	1.9
	Deceased wife	76	89.1
Educational level	Illiterate	91	33.7
	High school	66	24.4
	Diploma	50	18.5
	Associate and Bachelor	52	19.3
	Masters	11	4.1
Employment status	Employee	26	9.6
	Manual worker	6	2.2
	Self‐employed	25	9.3
	Retired	112	41.5
	Homemaker	87	32.2
	Unemployed	14	5.2
Urban/rural	Urban	212	78.5
	Rural	58	21.5
Residing in the provincial center	Yes	151	55.9
	No	119	44.1
Basic insurance	Yes	264	97.8
	No	6	2.2
Insurance type	Social security	199	75.4
	Health Service	49	18.6
	Other types of insurance	16	6.1
Supplemental Insurance	Yes	192	71.1
	no	78	28.9

More than half of the patients (51%) had ischemic (thrombotic) stroke, while strokes of unknown origin and hemorrhagic strokes accounted for 38% and 10%, respectively, in descending order of prevalence. Nearly half of the patients (47%) sought immediate medical attention and received a definitive diagnosis shortly after the onset of symptoms. However, more than half of them (53%) delayed seeking medical attention and diagnosis for at least 1 year despite experiencing symptoms. Most patients (98%) initiated necessary treatments immediately after a definitive diagnosis. However, 2% of patients delayed treatment for at least 1 year even after diagnosis. More than half of the patients (51%) are under continuous treatment by their treating physician, while a quarter (26%) receive treatment intermittently. In 95% of cases, neurologists bear the primary responsibility for patient care, and all patients receive necessary care simultaneously from both public and private healthcare centers. The most common post‐stroke complications reported were limb paralysis (26%), impaired consciousness (23%), and speech disorders (23%) (Table 2).

**TABLE 2:** Background characteristics related to the disease in patients.

Background characteristics	Grouping	Frequency	Percentage
Type of stroke	Ischemic	139	51.5
	Hemorrhagic	27	10
	Unknown	104	38.5
Time elapsed from the onset of symptoms to diagnosis	Immediately	128	47.4
	Less than 1 year	125	46.3
	1–4 years	17	6.3
Time elapsed from diagnosis to the start of care	Immediately	264	98.1
	Less than 1 year	3	1.1
	1–4 years	2	0.7
Monitoring status	Continuous	137	50.7
	Alternating	71	26.3
	In case of a severe problem	42	15.6
	Not being monitored	20	7.4
Treating physician	Neurologist	254	94.1
	Neurosurgeon	16	5.9
Place of care	Public centers	0	0
	Private centers	0	0
	A combination of private and public centers	270	100
Stroke complications	Facial paralysis	35	13
	Limb paralysis	70	25.9
	Speech disorders	62	23
	Impaired consciousness	63	23.3
	Others (numbness of hands and feet, bleeding eyes, etc.)	147	54.4

Patients with stroke pay an average annual amount of 339,580,000 IRR (915.86 USD) for receiving healthcare services, with 17,300,000 IRR (46.66 USD) allocated to diagnostic services (5%), 39,220,000 IRR (105.78 USD) to physician visits (12%), and 283,050,000 IRR (763.40 USD) to therapeutic care (83%). The most expensive diagnostic services include laboratory tests (47%), MRI scans (32%), and ultrasound (10%). Regarding physician visits, the highest costs are for neurology specialists (52%), followed by traditional medicine practitioners (32%). In terms of therapeutic expenses, the highest costs are associated with medication (38%), hospitalization (19%), and physiotherapy (16%) (Table 3).

**TABLE 3:** Direct medical costs imposed on patients.

Types of care costs		Min. IRR (USD)	Max. IRR (USD)	Mean IRR (USD)	St. dev. ± IRR (USD)
Diagnostic services	Laboratory	0	150,000,000 (404.55)	8,201,900 (22.12)	13,301,310 (35.87)
	Ultrasound	0	45,000,000 (121.36)	1,759,600 (4.74)	5,675,950 (15.31)
	CT Scan	0	3,000,000 (8.09)	86,300 (0.23)	383,100 (1.03)
	MRI	0	100,000,000 (269.70)	5,537,800 (14.93)	12,431,940 (33.53)
	Radiography	0	15,000,000 (40.45)	55,600 (0.15)	912,870 (2.46)
	Others (Nerve Conduction Study, Eye Imaging)	0	450,000,000 (1213.67)	1,666,700 (4.49)	27,386,130 (73.86)
	Total	0	460,000,000 (1240.64)	17,307,778 (46.68)	34,698,534.6 (93.58)
Visit	General Practitioner	0	7,500,000 (20.23)	358,900 (0.97)	788,710 (2.13)
	Internal Medicine/Gastroenterologist	0	15,000,000 (40.45)	1,548,100 (4.17)	2,759,340 (7.44)
	Neurologist	0	150,000,000 (404.55)	20,243,700 (54.60)	26,014,240 (70.16)
	Neurosurgeon	0	42,000,000 (113.27)	1,383,700 (3.3)	4,155,130 (11.20)
	Nutritionist	0	2,400,000,000 (6472.89)	2,979,600 (8.03)	15,155,650 (40.87)
	Traditional Medicine	0	240,000,000 (647.29)	12,560,400 (33.87)	33,558,830 (6.56)
	Others (Ophthalmologists, etc.)	0	40,000,000 (107.88)	148,100 (0.40)	2,434,320 (6.56)
	Total	0	280,000,000 (755.17)	39,222,593 (105.78)	47,280,298.6 (127.51)
Therapeutic services	Surgery	0	540,000,000 (1456.40)	8,670,400 (23.38)	49,740,730 (134.15)
	Hospitalization	0	1,080,000,000 (2912.80)	54,550,200 (147.12)	125,002,160 (337.13)
	Medication	0	360,000,000 (970.93)	107,307,400 (289.41)	70,715,190 (190.72)
	Dietary Supplements	0	540,000,000 (1456.40)	32,633,300 (88.01)	67,544,460 (182.17)
	Home Care	0	960,000,000 (2,589.16)	20,000,000 (53.94)	75,535,070 (203.72)
	Physiotherapy	0	360,000,000 (970.93)	44,064,800 (118.84)	77,108,290 (207.96)
	Occupational Therapy	0	420,000,000 (1132.75)	2,770,400 (7.47)	18,747,570 (50.56)
	Speech Therapy	0	300,000,000 (809.11)	13,055,600 (35.21)	34,529,780 (93.12)
	Total	0	1,638,000,000 (4417.75)	283,052,074 (763.40)	267525513.6 (72,152)
Total cost		0	1,759,000,000 (4744.09)	339,582,444 (915.86)	290556278.4 (783.64)

The analysis of patients' income and expenditure patterns indicates that their annual income amounts to 1,927,510,000 IRR (5198.57), with 442,180,000 IRR (1192.57) allocated to non‐food expenditures (23%) (Table 4). Based on this, the ratio of direct medical expenses to non‐food spending is estimated at 1.30 ( ± 2.30), and 67% of patients face health cost barriers.

**TABLE 4:** Annual income of patients and their families and their spending patterns.

Household income and expenses	Min. IRR (USD)	Max. IRR (USD)	Mean IRR (USD)	St. dev. ± IRR (USD)
Income	72,000,000 (194.18)	6,000,000,000 (16182.23)	1,927,518,500 (5198.59)	1,011,876,750 (2729.07)
Food expenditures	40,000,000 (107.88)	3,200,000,000 (8630.52)	1,354,955,600 (3654.37)	413,877,900 (1116.24)
Non‐food expenditures	32,000,000 (86.30)	2,800,000,000 (7551.71)	442,185,200 (1192.59)	365,234,610 (985.05)
Savings	0 (0)	1,500,000,000 (4045.56)	73,963,000 (199.48)	202,958,230 (547.38)

The study used regression modeling to investigate the relationship between demographic and background variables and the incidence of CHE in patients. Univariate regression analysis only showed a significant statistical relationship between the variables “limb paralysis” and “consciousness impairment” (as side effects of a stroke) and the incidence of CHE (*p* < 0.05). This analysis indicated that the occurrence of CHE in patients with limb paralysis and consciousness impairment is 86% and 74% higher, respectively, compared to patients without these complications. Multivariate regression analysis showed a significant relationship between variables such as age, supplemental insurance, limb paralysis, consciousness impairment, and development of other disease complications (*p* < 0.05). Specifically, the likelihood of incurring CHE increases by 62% for each additional 10 years of age in patients. Additionally, not having supplemental insurance leads to a 200% (three times) increase in the likelihood of incurring these costs. Furthermore, the occurrence of limb paralysis, consciousness impairment, and other disease complications increases the probability of incurring CHE by 87%, 74%, and 49%, respectively. In the final reduced model, age and having supplemental insurance, as well as facial paralysis, limb paralysis, consciousness impairment, and other disease complications, were identified as the main variables affecting the incidence of CHE. Specifically, each 10‐year increase in age results in a 57% increase in the chance of incurring these costs, and not having supplemental insurance increases the probability by 150% (2.5 times). Moreover, the occurrence of facial paralysis, limb paralysis, consciousness impairment, and other disease complications leads to increases of 75%, 88%, 78%, and 47%, respectively, in the probability of incurring CHE (Table 5).

**TABLE 5:** The statistical relationship between demographic and contextual variables with CHE.

Variable	Univariate Model		Multiple Model		Reduced Model			
	Non‐adjusted OR	*p* value	Adjusted OR	*p* value	Adjusted OR	95% CI for OR		*p* value
						Lower	Upper	
Age	1.545	0.0625	1.625	< 0.001	1.572	1.204	2.052	0.001
Gender	0.762	0.466						
Marital status	1.030	0.899						
Education	0.842	0.348						
Occupation	0.932	0.669						
Residence in the provincial capital	1.129	0.727						
Urban/Rural	1.048	0.925						
Having basic insurance	0.845	0.462						
Type of basic insurance	0.904	0.745						
Having supplemental insurance	2.368	0.058	3.085	0.006	2.501	1.173	5.334	0.018
Type of stroke	1.381	0.074	1.319	0.110				
The onset of symptoms to diagnosis	1.474	0.0154	0.312	0.174				
Diagnosis to care	0.360	0.270						
Being under observation	0.836	0.349						
Treatment provider	2.898	0.175	2.626	0.215				
Place of care	1.422	0.274						
Facial paralysis	0.357	0.138	0.290	0.064	0.253	0.069	0.934	0.039
Limb paralysis	0.145	0.001	0.134	< 0.001	0.122	0.041	0.362	< 0.001
Speech disorder	0.733	0.479						
Consciousness disorder	0.269	0.010	0.261	0.006	0.228	0.088	0.589	0.002
Other complications	0.576	0.120	0.509	0.035	0.534	0.289	0.988	0.045

## 4 Discussion

The study findings showed that strokes occur at younger ages due to the rising prevalence of their risk factors; this trend has been demonstrated in ischemic heart diseases, too [19]. One‐third of the patients are illiterate, and only one‐fourth have a university education, potentially compromising their health literacy and ability to engage in self‐care practices effectively. Most patients have basic health insurance, and over two‐thirds have supplemental health insurance. However, more than half of the patients, despite experiencing symptoms of the disease, have delayed seeking diagnosis and follow‐up for at least 1 year. Likewise, only half of the patients regularly receive care under the supervision of a treating physician, often a neurologist, highlighting the necessity for community education and improving accessibility to healthcare, both financially and geographically. This status indicates financial restrictions, inadequate health insurance coverage, and a lack of public understanding about the importance of stroke and its effects and consequences [16, 19].

Among the diagnostic services received, the highest proportion of expenses is attributed to laboratory tests, MRI scans, and ultrasounds. Visits to neurology specialists and traditional medicine practitioners account for the largest share of the visit costs. Given that most patients are under the care of neurologists, higher costs for their services are expected. However, the significant share of expenses allocated to services provided by traditional healers, who have no scientific connection to the disease, indicates major cultural issues, weak health literacy, and the prevalence of superstition. Medication, hospitalization, and physiotherapy costs constitute the highest medical care expenses. These findings highlight the reliance on imported medications and the inadequacy of rehabilitation services in the public sector [19].

Stroke patients spend an average of 339,580,000 (915.86) IRR annually on healthcare expenses, with diagnostic services, visits, and medical care comprising 5%, 12%, and 83%, respectively. The ratio of direct medical costs to non‐food expenses in patients is estimated at 1.30 ( ± 2.3), and the incidence of CHE is 67%. The main influencing factors in the statistical analysis of the significant correlation between demographic and background variables and the incidence of CHE were age, the presence of supplemental insurance, and the occurrence of disease complications like facial paralysis, limb paralysis, and consciousness impairment. Age affects expenses due to a higher likelihood of illness and related complications, leading to more complex and costly treatments in older individuals [19]. Supplemental insurance can be attributed to improved financial accessibility to healthcare services and enhanced illness management in insured individuals, reducing CHE [20]. The negative impact of disease complications can result in increased costs associated with managing these complications, prolonged treatment periods, and reduced economic capacity of patients [16].

The study by Lekander in 2017 in Sweden showed that the total direct and indirect costs imposed on stroke patients 1 year after the onset of the disease amounted to approximately 37,000 euros, and 2 years later, it was about 50,000 euros. Additionally, the annual costs imposed on patients varied depending on the severity of disability following stroke, ranging from 10,000 euros to 120,000 euros [21]. A study by Jennum in 2015 in Denmark demonstrated that the direct medical costs imposed on patients with hemorrhagic, ischemic, and unspecified types of stroke amounted to 10,720 euros, 8205 euros, and 7377 euros, respectively. Furthermore, the indirect costs due to reduced economic productivity of the patients' spouses for these three types of stroke were 989 euros, 1544 euros, and 1645 euros annually [11].

The study by Abdo in 2016 in Lebanon indicated that the daily cost of hospitalization due to stroke was approximately 538 dollars, with an average total hospitalization cost per patient during the treatment period amounting to 6961 dollars. Detailed investigations showed that 26.8% of the costs were attributed to hoteling, 22.3% to radiology, 15.7% to physician visits, 14.4% to laboratory services, 14.6% to medications, and 6.2% to other expenses. This study reported the direct hospitalization costs per patient in high‐income countries as 7130 dollars, 9688 dollars in the United States, 1917 dollars in neighboring countries like Turkey, and 1578 dollars in Pakistan [22]. Additionally, the Zhang study in 2013 in China calculated the hospitalization cost for stroke patients as 3212 dollars; in the United States in 2008, it was 20,396 dollars; in the Netherlands in 2012, it was 6845 dollars; in Sweden in 2001, it was 8104 dollars; in Singapore in 2012, it was 10,190 dollars; in South Korea in 2007 it was 8732 dollars, and in Japan in 2002 it was 8662 dollars [4].

The study by Luengo‐Fernandez in 2017 showed that stroke imposed approximately 60 billion euros on European countries in 2017, with 45% of these costs attributed to healthcare, equivalent to 1.7% of their total healthcare budget. This figure translates to 59 euros per European citizen, varying from 11 euros in Bulgaria to 140 euros in Finland [3]. Another study estimated the healthcare costs related to stroke per affected individual in the United States and European countries to be approximately 3875 euros and 3483 euros, respectively [3].

Therefore, executive recommendations include educating the public about the importance of stroke and its prevention. Developing and implementing stroke care packages is also advised. Improving basic and higher education literacy, as well as health literacy, in society is necessary. Implementing intervention programs can enhance the lifestyle of urban populations through environmental sanitation and the creation of sports and recreational facilities. Strengthening insurance systems based on universal health coverage principles is essential. Expanding healthcare services both quantitatively and qualitatively by the public sector is recommended. Establishing appropriate referral systems for necessary patient care is crucial. Emphasis should be placed on supervising patients by competent neurologists. Educating people to avoid superstitions is vital. The government can support pharmaceutical companies in producing indigenous drugs. Providing special social support for elderly patients is necessary. Government and insurance programs should offer special support for individuals with severe conditions, based on the principles of vertical equity, which means unequal treatment for unequal needs. The research team also suggests conducting similar studies on other highly prevalent and costly diseases. These studies can describe their status and help policymakers improve intervention strategies.

This study has several limitations. One limitation is its reliance on patient memory to recall incurred expenses, which increases the risk of recall bias. This limitation arises because there is no comprehensive health information system for patients to register their care and payments systematically. Another limitation is the inability to obtain responses from some patients due to poor health conditions or death before the study, which could lead to underestimating actual costs. These patients are often in more acute phases of the disease, requiring more extensive and complex care, which tends to be more specialized and costly, increasing the likelihood of encountering CHE in these individuals.

## 5 Conclusion

The findings of this study suggest that the financial burden faced by stroke patients is not proportionate when compared to similar studies conducted both domestically and internationally on other chronic disabling diseases, such as multiple sclerosis, ischemic heart and vascular diseases, COVID‐19, and gastrointestinal cancers. The high costs imposed on patients with strokes, especially in terms of medications and hospitalization, have imposed unusual economic pressure on patients and the occurrence of an unusual and unacceptable level of CHE. The study also shows that the patient's age, having supplementary health insurance, and the level of patient exposure to numerous complications of stroke are the main factors in the occurrence of CHE. There is an urgent need to develop and implement targeted and effective interventions. Researchers aim to raise awareness among decision‐makers, describe the situation, and suggest options for improvement. The results of this study can be used as a valuable resource for healthcare managers and policymakers.

## Author Contributions

**Sayed Amir Mortazavi:** conceptualization, software, writing – original draft, formal analysis, validation, investigation, **Sayed Saeed Kassaeian:** conceptualization, methodology, validation, writing – review and editing, formal analysis, **Navid Danaei:** conceptualization, methodology, software, writing – review and editing, data curation, validation, **Farid Gharibi:** conceptualization, methodology, software, validation, supervision, investigation, formal analysis, writing – original draft.

## Conflicts of Interest

The authors declare no conflicts of interest.

## Transparency Statement

The lead author Farid Gharibi affirms that this manuscript is an honest, accurate, and transparent account of the study being reported; that no important aspects of the study have been omitted; and that any discrepancies from the study as planned (and, if relevant, registered) have been explained.

## Data Availability

Data are stored at the origin of the university of study. If any researcher is interested, for a valid reason, they may contact the corresponding author (Farid Gharibi), gharibihsa@gmail.com. All authors have read and approved the final version of the manuscript. Farid Gharibi (corresponding author) had full access to all of the data in this study and takes complete responsibility for the integrity of the data and the accuracy of the data analysis. Farid Gharibi affirms that this manuscript is an honest, accurate, and transparent account of the study being reported, that no important aspects of the study have been omitted, and that any discrepancies from the study as planned (and, if relevant, registered) have been explained. The data that support the findings of this study are available on request from the corresponding author. The data are not publicly available due to privacy or ethical restrictions.
